# History of Illicit Stimulant Use Is Not Associated with Long-Lasting Changes in Learning of Fine Motor Skills in Humans

**DOI:** 10.1155/2016/9485079

**Published:** 2015-12-27

**Authors:** Gabrielle Todd, Verity Pearson-Dennett, Stanley C. Flavel, Miranda Haberfield, Hannah Edwards, Jason M. White

**Affiliations:** ^1^School of Pharmacy and Medical Sciences, University of South Australia, Adelaide, SA 5000, Australia; ^2^Sansom Institute for Health Research, University of South Australia, Adelaide, SA 5000, Australia

## Abstract

Little is known about the long-lasting effect of use of illicit stimulant drugs on learning of new motor skills. We hypothesised that abstinent individuals with a history of primarily methamphetamine and ecstasy use would exhibit normal learning of a visuomotor tracking task compared to controls. The study involved three groups: abstinent stimulant users (*n* = 21; 27 ± 6 yrs) and two gender-matched control groups comprising nondrug users (*n* = 16; 22 ± 4 yrs) and cannabis users (*n* = 16; 23 ± 5 yrs). Motor learning was assessed with a three-minute visuomotor tracking task. Subjects were instructed to follow a moving target on a computer screen with movement of the index finger. Metacarpophalangeal joint angle and first dorsal interosseous electromyographic activity were recorded. Pattern matching was assessed by cross-correlation of the joint angle and target traces. Distance from the target (tracking error) was also calculated. Motor learning was evident in the visuomotor task. Pattern matching improved over time (cross-correlation coefficient) and tracking error decreased. However, task performance did not differ between the groups. The results suggest that learning of a new fine visuomotor skill is unchanged in individuals with a history of illicit stimulant use.

## 1. Introduction

Learning of fine motor skills in humans is commonly investigated with tasks that involve visually guided movements of the hand (i.e., visuomotor tasks) [1, 2]. Tasks that involve tracking a moving target on a computer screen with movements of the hand have been particularly well characterised. Learning of such tasks is evidenced by an increase in the accuracy of pattern matching and a decrease in tracking error (i.e., deviation from the target line) over time (e.g., [3, 4]). Mechanisms that are thought to underlie learning of visuomotor tasks include changes in synaptic efficacy and functional reorganisation (plasticity) within the motor cortex (for review [5]).

Acute changes in the performance of visuomotor tasks have been observed following use of illicit drugs [6–9]. For example, the ability to track a moving target on a computer screen with movements of the hand is impaired for up to seven hours after cannabis use [6–8] (cf. [10]). Conversely, performance of a task that involves use of a joystick to keep a cursor centred in a target area (critical tracking test) is improved two hours after administration of 75 mg of 3,4-methylenedioxymethamphetamine (MDMA or “ecstasy”) in adults with a history of illicit drug use [9]. However, very little is known about the acute and long-lasting effect of illicit drug use on* learning* of these tasks.

Stimulant drugs such as amphetamine, methamphetamine, and/or cocaine have the greatest potential to affect learning of fine motor skills. These drugs cause acute accumulation of primarily dopamine in the synaptic cleft (for review [11, 12]) and their use has been shown to modulate plasticity in animals [13] and in the human motor cortex [14, 15]. For example, administration of a single therapeutic dose of amphetamine enhances use- (practice-) dependant plasticity in healthy adults [14, 15] and in some stroke patients [16]. Similar findings have also been observed following administration of a single dose of levodopa [17], a drug that promotes the synthesis of dopamine.

The relationship between amphetamine and use-dependent plasticity is likely to vary in a dose-dependent manner. In rodent prefrontal cortex, injection of a low-dose of amphetamine (0.1 mg/kg) results in acute enhancement of long-term potentiation whereas high doses (10 mg/kg) abolish long-term potentiation [13]. Furthermore, high doses of amphetamine or methamphetamine, like those used illicitly, are toxic to dopaminergic neurons (for review [11, 12]). Long-lasting changes in the human motor cortex and other movement-related brain regions have also been observed in individuals with a history of illicit stimulant use [18–20]. However, it is unclear if these long-lasting pathophysiological changes alter the ability of individuals to learn new fine motor skills. Preliminary evidence suggests that motor skill learning may be unaffected in the longer term given that individuals with a history of mixed illicit stimulant use can improve their performance on the grooved pegboard test across trials and adaptation of grip force during repeated lifting of a novel object has been observed in this population [21]. Furthermore, learning of a visuomotor tracking task (pursuit rotor) was not impaired in cocaine-dependent individuals undergoing detoxification during a 21-day inpatient substance abuse treatment program [22].

The aim of the current study was to further investigate the long-lasting effect of illicit stimulant use on learning of fine motor skills. The novel aspects of the current study include (a) inclusion of a stimulant-using population with a history of primarily methamphetamine and ecstasy use, (b) inclusion of a stimulant-using population that was not undergoing detoxification, (c) inclusion of a cannabis control group to differentiate the long-lasting effects of stimulant use from cannabis use, (d) quantification of lifetime drug history for all classes of licit and illicit drugs (rather than just the drug of interest), and (e) quantification of the magnitude of pattern matching and delay between movement of the target and movement of the finger with the use of cross-correlation analysis. We hypothesised that individuals with a history of illicit stimulant use would exhibit normal performance and learning of the visuomotor tracking task compared to two control groups (nondrug using group and cannabis using group). The hypothesis was based on normal learning of the visuomotor tracking task in adults undergoing cocaine detoxification [22], a population with a high prevalence of poly-stimulant use [23].

## 2. Materials and Methods

Motor learning was assessed in three groups of adults. The groups were gender-matched and comprised of 22 individuals with a history of illicit stimulant use (target “stimulant” group), 17 individuals with a history of cannabis use but not illicit stimulant use (positive “cannabis” control group), and 17 individuals with no history of illicit drug use (negative “nondrug” control group). The characteristics of each group are presented in Table 1. General inclusion criteria were being aged 18–50 yrs and right hand dominant (defined as a laterality quotient of >0.4 on the Edinburgh Handedness Inventory [24]). Additional inclusion criteria for the stimulant group were use of amphetamine, methamphetamine, ecstasy, and/or cocaine on greater than 10 occasions. Additional inclusion criteria for the cannabis group were use of cannabis on greater than 10 occasions but no history of stimulant use. The cannabis group acted as a positive control group to ensure that any observed changes in motor learning were not the result of cannabis use given that cannabis use is common among stimulant users. The additional inclusion criteria for the control group was no history of illicit drug use (alcohol and tobacco use were permitted). All experimental procedures were approved by the Human Research Ethics Committee at the University of South Australia and conducted according to The Declaration of Helsinki. Written informed consent was obtained prior to participation.

### 2.1. Subject Screening

Subjects underwent a series of screening tests prior to the experiment. Subjects were asked to provide a urine sample for routine drug screening (PSCupA-6MBAU, US Diagnostics Inc., Huntsville, Alabama, USA) and to complete a brief medical history questionnaire [25]. Subjects were then interviewed about their lifetime and recent use of alcohol, tobacco, and illicit drugs. The interview was guided by an in-house questionnaire that listed 20 illicit drugs and requested information on other illicit drugs not listed. Items on the questionnaire included age of first use, age of regular use, duration of use, frequency of use (current and lifetime), average dose (current and lifetime), frequency of high dose use, and time since last use defined for each drug. The number of drug overdoses and treatment for drug dependency were also noted. The final screening test involved a neuropsychological assessment of memory and cognition. Four cognitive domains were assessed. New learning was assessed with Logical Memory I and II [26], executive functioning was assessed with Verbal Trails and Verbal Fluency [27, 28], working memory was assessed with Digit Span backwards [29], and attention was assessed with Digit Span forwards [29].

Common exclusion criteria across the groups included (a) history of neurological damage and/or neurological illness prior to illicit drug use, (b) current use of prescribed medications that act on the nervous system (e.g., antidepressants), (c) frequent illicit opioid use (>5 times), and (d) positive urine test for amphetamine, methamphetamine, MDMA, cocaine, opioids, and/or benzodiazepines. Subjects who tested positive for cannabis were allowed to participate if use was >12 hours prior to the experiment. This exemption was due to the metabolite of the main active ingredient of cannabis (tetrahydrocannabinol) remaining in the body for up to 80 days after last use (for review [30]).

### 2.2. Experimental Protocol

The experiment began with preparation and positioning of two surface electromyographic (EMG) electrodes (Ag-AgCl, 10 mm diameter) over the muscle belly and tendon of the right first dorsal interosseus muscle. EMG activity was amplified (300 or 1000x), filtered (20–1000 Hz), and sampled at 2000 Hz using a data acquisition system (1902 with Power 1401 Interface and Signal and Spike2 software: Cambridge Electronic Design, Cambridge, UK). Subjects then completed three tasks with the right (dominant) hand.

**Figure 1:**
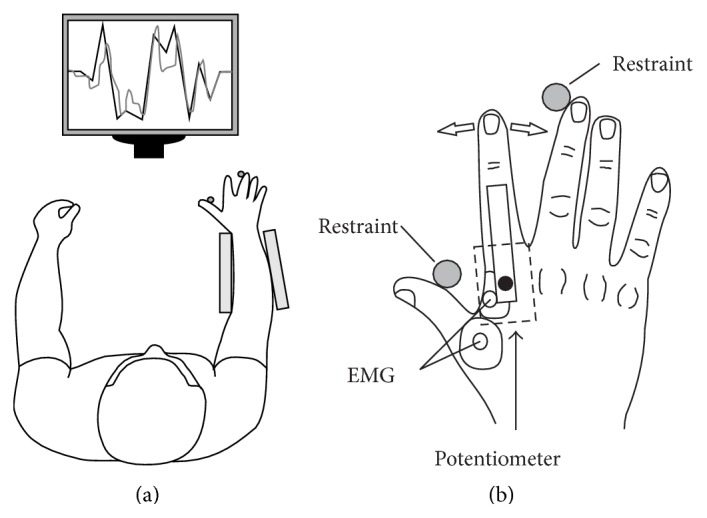
Experimental apparatus for the visuomotor tracking task. (a) Subjects were instructed to match their index finger metacarpophalangeal (MCP) joint angle with a moving target displayed on a computer screen. The target moved across the screen while making unpredictable upward and downward movements. Abduction of the finger moved the feedback line downward while adduction moved the feedback line upward. The maximum MCP joint angle movement was ±10° from neutral. (b) Medial-lateral movement of the index finger was recorded with a potentiometer. The axis of rotation of the potentiometer was positioned over the MCP joint. Muscle activity was also recorded from the first dorsal interosseous muscle using surface EMG.

**Figure 2:**
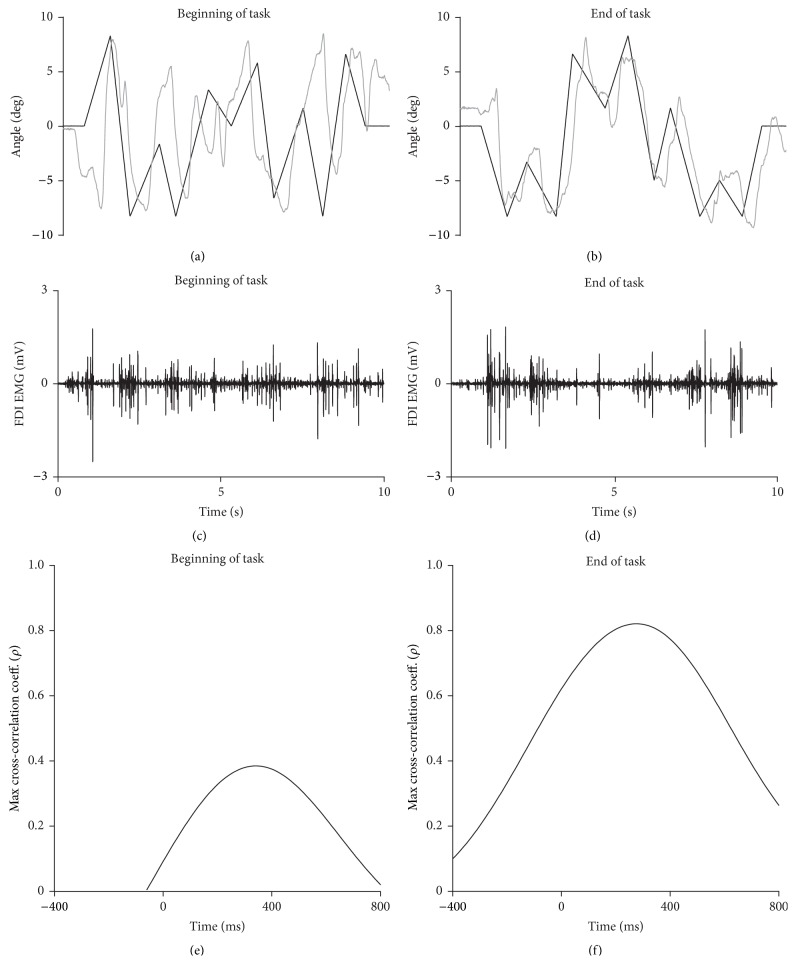
Single-subject data for the three-minute visuomotor tracking task. Data are from an individual in the stimulant group. (a) and (b) Raw metacarpophalangeal joint angle trace (grey line) and target line (black line) at the beginning of the first (0–30 s) and last (150–180 s) epoch, respectively. (c) and (d) Raw EMG traces for the accompanying time frame. (e) and (f) Cross-correlogram for the same subject for the first (0–30 s) and last (150–180 s) epoch, respectively.

The first task involved a three-minute visuomotor tracking task to assess motor learning. Subjects were instructed to match their index finger metacarpophalangeal (MCP) joint angle with a moving target displayed on a 22-inch computer monitor (P2210 Flat Panel Monitor, Dell Inc.). The screen was positioned two metres in front of the subject's chest and both the target and MCP joint angle were displayed (Figure 1(a)) as a solid red line on a white background. The moving target consisted of 18 unique 10 s frames (see Figures 2(a) and 2(b)) and subjects were instructed to follow the target as closely as possible. The target moved across the screen while making unpredictable upward and downward movements. Abduction of the finger moved the feedback line downward while adduction moved the feedback line upward. The maximum MCP joint angle movement was ±10° from neutral and the thumb and middle finger were restrained (Figure 1(b)). Medial-lateral movement of the index finger was recorded with a potentiometer (model 157, Vishay, NSW, Australia), with the axis of rotation positioned over the MCP joint (Figure 1(b)). The potentiometer signal was filtered (DC-100 Hz) and sampled at 2,000 Hz using the same data acquisition system.

The second task involved a brief (2-3 s) maximal isometric abduction of the index finger for normalisation of voluntary EMG recorded during the visuomotor tracking task. Three brief maximal voluntary contractions (MVCs) were performed and each contraction was separated by approximately one minute of rest to avoid fatigue. Verbal encouragement and visual feedback of force were provided. Force was recorded using a linear strain gauge (LC1205-K020, A&D Co. Ltd., Tokyo, Japan) positioned at the proximal interphalangeal joint. The thumb and middle finger were restrained. Force was recorded using the above mentioned data acquisition system. Force signals were amplified (1000x), filtered (DC-100 Hz), and sampled at 400 Hz. The EMG electrodes were removed after the last MVC.

The third task involved assessment of motor learning with the use of the Grooved Pegboard test (model 32025, Lafayette Instrument, Lafayette, IN, USA). The test involves placing 25 key-shaped pegs into corresponding grooves. Subjects were instructed to complete the task (one pin at a time) as fast as possible and in a set sequence. The time taken to complete the test was recorded. Three trials were performed and each trial was separated by one minute of rest to avoid fatigue.

Factors that could alter performance of the above tasks include speed of information processing and/or symptoms of depression. These factors were assessed in the fourth and fifth tasks. Symptoms of depression (over the past two weeks) were assessed with a 21-item self-report rating scale (Beck Depression Inventory-II, [31]) and speed of information processing was assessed with the inspection time test. The inspection time test involves presentation of two parallel lines on a computer screen and indicating which of the two lines was shorter [32]. The minimum exposure time required to accurately determine the shorter line was recorded. The test is a measure of speed and efficiency of information processing independent of the motor component of reaction time.

### 2.3. Data Analysis

Performance on the visuomotor tracking task was assessed in 30-second epochs for each subject. The absolute difference between the target angle and MCP joint angle at each sample point was measured (“tracking error”) and averaged over the 30 s epoch. The target angle was also cross-correlated with the MCP angle and the maximum cross-correlation coefficient and the lag time to achieve the maximum cross-correlation coefficient were determined (e.g., see Figures 2(e) and 2(f)). Root mean square (RMS) EMG activity was measured during the task (e.g., see Figures 2(c) and 2(d)) and expressed as a percentage of the average RMS EMG measured during the brief maximal contractions.

Group data are presented as the mean ± standard deviation (SD) in the text and mean ± standard error of the mean (SEM) in figures. Between-group comparison of subject characteristics (age, years of education), neuropsychological parameters, symptoms of depression (BDI-II score), speed of processing (inspection time), and lifetime use of alcohol (estimated total drinks) and tobacco (estimated total cigarettes) was made with one-way analysis of variance (ANOVA). Nonparametric data were transformed to ranks and a one-way ANOVA on ranks was performed (SigmaPlot for Windows Version 11.0, Systat Software Inc., San Jose, USA). Data for the visuomotor task and grooved pegboard test were analysed with two-way repeated measures ANOVA for comparison of group (between-subject factor) and time (within-subject factor). This analysis was repeated with age as a covariate. Mauchly's test of sphericity was performed and the Greenhouse-Geisser method was used to correct for nonsphericity (IBM SPSS Statistics 20, Armonk NY, USA). Post hoc discrimination between means was made with the Student-Newman Keuls procedure. Unpaired Student's *t*-test was used to compare lifetime cannabis use (occasions) between the stimulant and cannabis groups. Paired Student's *t*-test was used to compare lifetime use of ecstasy and amphetamine-like stimulants within the stimulant group. Pearson Product Moment or Spearman Rank Order correlation was used to investigate the relationship between drug-use characteristics and (a) learning (change) and (b) endpoint performance of the visuomotor task and grooved pegboard test. The relation between duration of abstinence from stimulants and the change in tracking error was also investigated with linear regression analysis (SigmaPlot for Windows Version 11.0, Systat Software Inc., San Jose, USA). Significance was set at *P* < 0.05.

## 3. Results

### 3.1. Subject Characteristics

One control subject, one cannabis subject, and one stimulant subject were unable to perform the visuomotor tracking task and their data was omitted from further analysis. The characteristics of the remaining 53 subjects are presented in Table 1. The groups significantly differed in age (*F*_2,50_ = 4.758, *P* = 0.013) but not in years of education, laterality quotient (index of hand dominance), or performance on the neuropsychological tests of memory and cognition. The average age of the stimulant group was 4-5 yrs older than the control and cannabis groups (*P* < 0.028) but the average age of the control and cannabis groups did not differ from one another.

### 3.2. Drug History

Use of alcohol and tobacco significantly differed between the groups (alcohol: *F*_2,48_ = 51.043; *P* < 0.001, tobacco: *F*_2,50_ = 35.707; *P* < 0.001). Lifetime use of alcohol (estimated total drinks) and tobacco (estimated total cigarettes) was greatest in the stimulant group and least in the control group (*P* < 0.001, Table 1).

Table 2 shows the percentage of subjects within each group that had used various classes of illicit drugs. In the stimulant group, ecstasy was the most commonly used stimulant followed by methamphetamine, cocaine, and recreational use of pharmaceutical stimulants. Polydrug use was common in the stimulant group and less common in the cannabis group. All subjects in the stimulant group had used cannabis and the majority of subjects had used hallucinogens (primarily lysergic acid diethylamide or “LSD” and “magic” mushrooms) and inhalants (primarily nitrous oxide). Illicit use of sedatives and opioids was uncommon and total lifetime use of these drugs was low in the stimulant group (sedatives: 15 ± 30 occasions; opioids: 3 ± 1 occasions).

Table 3 shows single subject and group data for lifetime use of amphetamine-like stimulants, ecstasy, and cannabis in the stimulant group. Lifetime use of amphetamine-like stimulants was greater than lifetime use of ecstasy. The average duration of abstinence from stimulants was 1.6 ± 3.0 yrs (range: 7 days–12 yrs). Lifetime use of cannabis was significantly greater in the stimulant group (1,655 ± 2,061 occasions) than in the cannabis group (222 ± 334 occasions; *P* = 0.009) and the average duration of abstinence from cannabis was 224 ± 635 days (range: 1 day–8 yrs) and 395 ± 617 days (range: 1 day–5 yrs) for each group, respectively. No drug overdoses were reported in the control and cannabis groups, but five subjects in the stimulant group reported having experienced a drug overdose.

### 3.3. Visuomotor Tracking

Figures 2(a) and 2(b) show raw traces of MCP joint angle from a single subject in the stimulant group at the beginning and end of the visuomotor task. At the beginning of the task (first epoch: 0–30 s), the MCP joint angle trace partially resembled the target pattern (maximal cross-correlation coefficient: 0.39, lag time: 340 ms, tracking error: 4.4°; Figure 2(e)). Performance improved over time evidenced by a greater maximal cross-correlation coefficient (0.82) and reduced tracking error (2.7°) and lag time (280 ms) in the final epoch (150–180 s; Figure 2(f)).

**Figure 3:**
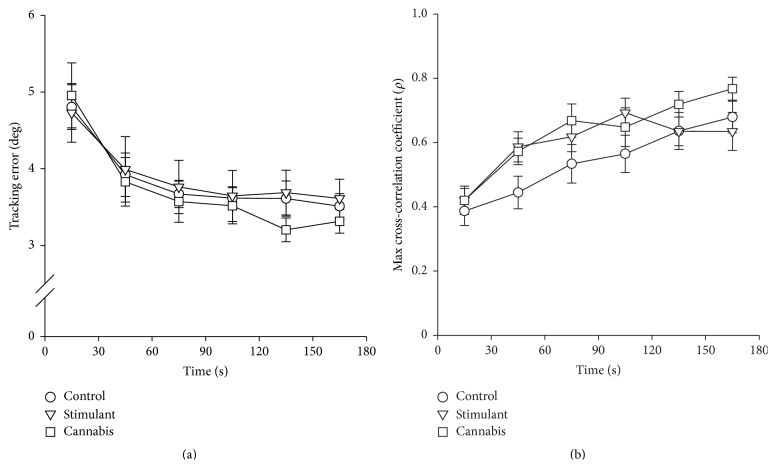
Group data showing performance during the visuomotor tracking task. (a) Tracking error. (b) Maximum cross-correlation coefficient (derived from cross-correlation of the target angle with metacarpophalangeal joint angle). Data for the control (circles), stimulant (triangles), and cannabis (squares) groups are shown.

Figure 3 shows group data for the visuomotor tracking task. There was a significant main effect of time on the absolute difference between the target angle and MCP angle (i.e., tracking error, *F*_5,250_ = 30.687, *P* < 0.001; Figure 3(a)). Tracking error significantly decreased over the first minute of the task (from epoch 1 to epoch 2, *P* < 0.044) but remained unchanged thereafter. However, tracking error did not differ between the groups and there was no significant group-by-time interaction.

The accuracy of pattern matching between the target angle and MCP joint angle was assessed for each subject by cross-correlation of the target angle with the MCP joint angle. A cross-correlation coefficient of 1 would indicate a perfect match whereas a cross-correlation coefficient of 0 would indicate no match. Across the groups, there was a significant main effect of time on the maximum cross-correlation coefficient (*F*_5,250_ = 43.770, *P* < 0.001, Figure 3(b)). The maximum cross-correlation coefficient did not significantly differ between groups, but there was a significant group-by-time interaction (*F*_10,250_ = 2.684, *P* = 0.010). The interaction arose from a subtle difference in the timing of improvement and attainment of a plateau in performance between groups, but not in the magnitude of improvement. In the stimulant group, the maximum cross-correlation increased over the first minute of the task (from epoch 1 to epoch 2) but remained relatively unchanged thereafter (*P* < 0.001, except for the epoch 2 versus epoch 4 comparison: *P* = 0.029). In the control and cannabis groups, the maximum cross-correlation coefficient increased for longer, up until 2–2.5 mins into the task (from epoch 1 to epochs 4-5, *P* < 0.039).

Voluntary RMS EMG (% of maximum) did not significantly differ over time or between groups (average across epochs: control = 11.0 ± 6.7%, stimulant = 10.7 ± 3.8%, and cannabis = 14.4 ± 10.8%; data not shown). The lag time between movement of the target and movement of the MCP joint angle also did not significantly differ over time or between groups (average across epochs: control = 302 ± 298 ms, stimulant = 423 ± 232 ms, and cannabis = 351 ± 221 ms; data not shown). There was also no significant group-by-time interaction on voluntary RMS EMG or lag time.

The analysis of each parameter in the visuomotor tracking task was repeated with age as a covariate. No significant main effect of group was observed after accounting for age.

**Figure 4:**
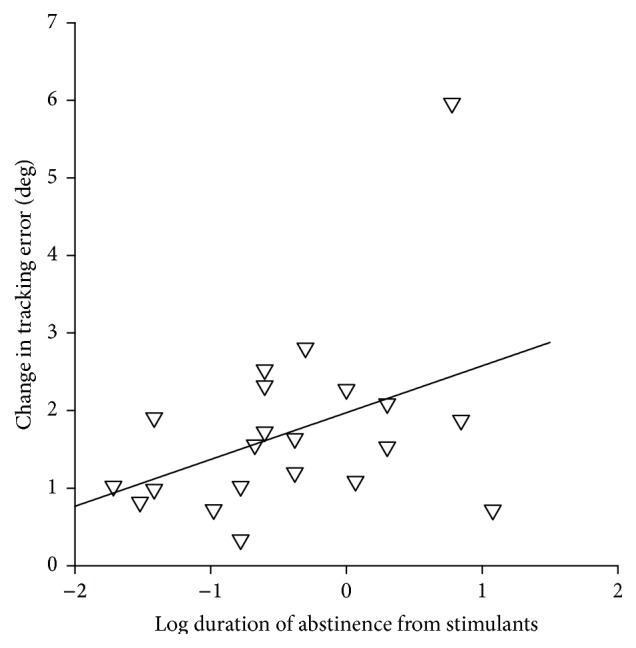
Correlation between duration of abstinence from stimulants and change in the tracking error across the visuomotor task. Single subject data for the stimulant group are shown and log duration of abstinence is plotted on the *x*-axis. The correlation did not reach statistical significance (*r* = 0.383, *P* = 0.085). Solid line shows the result of a linear regression analysis (*P* = 0.070).

In the stimulant group, the relation between stimulant drug use characteristics (total lifetime use or duration of abstinence) and learning and endpoint performance (epoch 6) on the visuomotor tracking task was explored with correlation analyses. No significant correlations were observed. However, there was a trend for a correlation between duration of abstinence from stimulants and change (reduction) in tracking error across the visuomotor task (*r* = 0.383, *P* = 0.085). Individuals with a shorter duration of abstinence tended to exhibit less change in tracking error (i.e., less improvement) than individuals with a longer duration of abstinence (Figure 4). In the cannabis group, there was no significant correlation between cannabis drug use characteristics and learning and endpoint performance of the visuomotor task.

### 3.4. Grooved Pegboard

**Figure 5:**
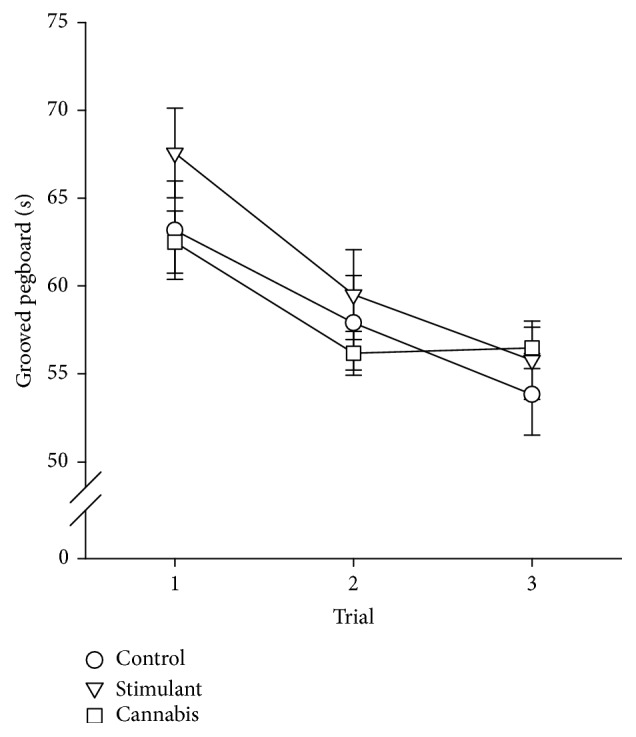
Group data showing performance during the grooved pegboard test. Data for the control (circles), stimulant (triangles), and cannabis (squares) groups are shown.

Figure 5 shows group data for the grooved pegboard test. There was a significant main effect of trial on the grooved pegboard test (*F*_2,100_ = 104.837, *P* < 0.001). Performance time significantly decreased across trials, indicating an improvement in performance. There was no significant main effect of group on the grooved pegboard test, but there was a significant group-by-trial interaction (*F*_4,100_ = 4.391, *P* = 0.003). Performance improvement was evident across all three trials in the control and stimulant groups (*P* < 0.008), but improvement was only observed between trials one and two in the cannabis group (*P* < 0.001). There was no significant correlation between stimulant and cannabis drug use characteristics (total lifetime use or duration of abstinence) and learning and endpoint performance (trial three) on the grooved pegboard test in the stimulant and cannabis groups, respectively. Analysis of the grooved pegboard data was repeated with age as a covariate. No significant main effect of group was observed after accounting for age.

### 3.5. Symptoms of Depression and Speed of Information Processing

Symptoms of depression (BDI-II score) significantly differed between the groups (*F*_2,50_ = 7.352, *P* = 0.002). As expected, subjects in the stimulant group (*P* = 0.001) and cannabis group (*P* = 0.026) experienced significantly more symptoms of depression than subjects in the nondrug using control group (Table 1). Three subjects in the stimulant group and one subject in the cannabis group had received a formal diagnosis of depression (after commencing illicit drug use), but none were being medicated at the time of the experiment. The groups did not significantly differ in speed of information processing (inspection time, Table 1).

## 4. Discussion

Performance of the visuomotor tracking task and grooved pegboard test improved over time. The improvement in performance is indicative of motor learning. We have shown for the first time that individuals with a history of illicit stimulant use exhibit (a) normal performance of a visuomotor tracking task and (b) normal learning of fine motor skills. The latter finding supports our initial hypothesis.

Performance of the visuomotor tracking task requires an awareness of where the index finger is in space (proprioception) and relative to the target as well as integration of visual input and motor output. Sensory feedback from the periphery and ongoing modulation of movement are also important. The lack of a between-group difference in performance of the visuomotor tracking task suggests that use of illicit stimulants drugs is not associated with long-lasting changes in the physiology that underlies performance of this task. Alternatively, movement-related brain regions may employ compensatory mechanisms that are capable of overcoming any drug-related deficits.

Learning of the visuomotor tracking task occurred across groups. Learning was evidenced by an increase in the accuracy of pattern matching (i.e., increase in the cross-correlation coefficient) and a decrease in the tracking error (i.e., difference between the target and MCP joint angle) over time. The majority of the improvement was observed in the first 90 s of the task with a plateau in performance observed thereafter. The improvement in performance did not appear to be associated with changes in first dorsal interosseous muscle activity or the lag between movement of the target and movement of the MCP joint because these parameters did not significantly change over time.

Learning of the visuomotor tracking task did not differ between groups. This suggests that learning of visuomotor tasks that involve fine movements of the hand is unaffected in individuals with a history of illicit stimulant use. Lifetime use of illicit stimulants was high in the stimulant group (271 ± 513 occasions) so the existence of any long-lasting effects of illicit stimulant use on learning should have been apparent in this group. However, subjects in the stimulant group had been abstinent from stimulants for an average of 1.6 ± 3.0 yrs (range: 7 days–12 yrs) and any deficits in learning and/or task performance could have recovered during the weeks or months following cessation of use. The trend for a positive correlation between duration of abstinence from stimulants and change in tracking error supports this view (*P* = 0.085, Figure 4). However, a larger sample size would be required to confirm this.

The sensitivity of the equipment and data analysis was sufficient to observe a between-group difference if one had been present. The methodology enabled detection of a very small improvement in tracking error, of as little as one degree between the joint angle and target angle, during the first minute of the visuomotor tracking task and this small improvement was statistically significant. Thus, detection of a between-group difference of as little as one degree would have been measurable if it had been present but this was not the case.

The current study is the first to demonstrate normal performance and learning of a visuomotor tracking task in individuals with a history of illicit stimulant use. Normal performance of another fine motor skill, the grooved pegboard test, has been previously reported in individuals with a history of mixed stimulant use and confirmed in the current study [33–36]. However, there have also been reports of slower performance time on the grooved pegboard test in this population [20, 37–41], but the impairment became less apparent with increasing duration of drug abstinence [38]. Cannabis users who have abstained from cannabis use for a long period of time (52 ± 17 months) also exhibit normal performance of the grooved pegboard test [42]. We have added to the literature on this topic by investigating both performance and learning of the grooved pegboard task. The time to complete the grooved pegboard test decreased across trials, suggesting an improvement in performance and thus learning of the task. A significant group-by-trial interaction was also observed. Performance improvement was evident across all three trials in the control and stimulant groups, but improvement was only observed between trials one and two in the cannabis group.

The lack of an association between history of illicit stimulant use and performance and learning of fine motor skills is surprising given that long-lasting changes in movement-related brain regions have been observed in this population. Studies involving noninvasive brain stimulation show that history of predominantly methamphetamine and ecstasy use is associated with a long-lasting increase in excitability of the motor cortex and corticospinal pathway [18]. Changes in excitability of this pathway have also been observed in abstinent cocaine-dependent individuals [43, 44]. Neuroimaging studies also show reduced dopamine reuptake transporter [45] and dopamine (D2) receptor availability in the striatum of abstinent methamphetamine users [46]. Furthermore, abnormal morphology of the substantia nigra, a brain region with a high density of dopaminergic neurons, has also been observed in individuals with a history of primarily methamphetamine and ecstasy use [19]. The latter abnormality is a strong risk factor for developing Parkinson's disease later in life [47].

Factors other than illicit stimulant use have the potential to affect learning and performance of fine motor skills. Neuropsychological factors can influence learning and performance but are unlikely to have affected the results of the current study. No between-group differences were observed in the score on the inspection time test (speed of information processing) or tests of memory and cognition. Group data for these tests were also above (Logical Memory I and II, Verbal Trails, and Digit Span) or within one standard deviation (Verbal Fluency) of published normative data for the general population [48–51]. Attention and symptoms of depression are also unlikely to have influenced the results of the current study. The duration of each motor task was short (1–3 mins) and performance on the Digit Span forwards test (attention) did not differ between groups. The drug-using groups exhibited more symptoms of depression than the nondrug using group, but the groups did not differ in learning or performance of the tasks.

Other factors that could influence learning and performance of fine motor skills include age, gender, and lifetime use of alcohol, tobacco, and/or cannabis. Gender and cannabis use were accounted for in the experimental design. However, the possible effects of age and lifetime use of alcohol and tobacco were not accounted for because these parameters were not adequately matched between groups. The age of subjects in the stimulant group (27 ± 6 yrs) was on average 4-5 yrs older than subjects in the positive (cannabis: 23 ± 5 yrs) and negative (nondrug: 22 ± 4 yrs) control groups. The small age difference between stimulant and cannabis users is to be expected based on published epidemiological data on drug use patterns. In Australia, the prevalence of cannabis and stimulant use is highest in adults aged 20–29 yrs (21.3% and 5.9–9.9%, resp.), but the onset of cannabis use tends to occur at an earlier age than the onset of stimulant use (prevalence in individuals aged 14–19 yrs is 21.5% and 4.7–6.2%, resp.) [52]. The mean age of the groups matched the epidemiological data implying that the study sample was representative of the wider drug-using population. The small age difference between the groups is unlikely to have affected the results of the current study because no between-group differences in performance or learning were observed and the magnitude of learning associated with a comparable visuomotor task is similar for young (aged 20–35 yrs) and older (aged 60–75 yrs) adults [3]. The greater lifetime use of alcohol and tobacco in the drug using groups is also to be expected based on published epidemiological data. Individuals who use stimulants and/or cannabis are well known to consume more alcohol and tobacco than individuals with no history of illicit drug use [52, 53]. The lack of a between group difference in performance and learning of fine motor skills in the current study suggests that the greater lifetime use of alcohol and tobacco had a minimal effect on the outcome of the study.

In summary, the results of the current study suggest that history of illicit stimulant use is not associated with long-lasting changes in the learning and performance of fine visuomotor skills. The results of the current study have implications for rehabilitation of movement deficits in this population. Individuals with a history of illicit stimulant use are capable of learning new fine motor skills if this were required in a rehabilitation program. The results of the current study also have implications for the potential use of amphetamine as a therapeutic aid for stroke rehabilitation.

## Figures and Tables

**Table 1:** Subject characteristics for the control, stimulant, and cannabis groups.

	Control (*n* = 16)	Stimulant (*n* = 21)	Cannabis (*n* = 16)
Age (yrs)	22 ± 4	27 ± 6^*∗*§^	23 ± 5
Gender	10 M, 6 F	13 M, 8 F	10 M, 6 F
Laterality quotient	0.80 ± 0.16	0.84 ± 0.15	0.82 ± 0.12
Education (yrs)	15 ± 2	15 ± 2	16 ± 3
BDI-II score	2 ± 2	9 ± 7^*∗*^	6 ± 6^*∗*^
Inspection time (s)	0.73 ± 0.26	0.67 ± 0.19	0.70 ± 0.12
Lifetime alcohol use (total drinks)	55 ± 94	7,718 ± 7,236^*∗*§^	2,113 ± 2,936^*∗*^
Lifetime tobacco use (total cigarettes)	1 ± 2	26,943 ± 37,725^*∗*§^	1,648 ± 4,490^*∗*^

Data are mean ± standard deviation. ^*∗*^Significantly different from control group (*P* < 0.05). ^§^Significant difference between stimulant group and cannabis group (*P* < 0.05).

**Table 2:** Classes of illicit drugs consumed in the stimulant and cannabis groups.

	Stimulant group	Cannabis group
Stimulants	100%	0%
Ecstasy	100%	0%
Methamphetamine	81%	0%
Cocaine	57%	0%
Pharmaceutical	14%	0%
Cannabis	100%	100%
Hallucinogens	86%	31%
Inhalants	57%	6%
Sedatives	24%	0%
Opioids	29%	0%

Data are percentage of subjects that have consumed that class of illicit drug in their lifetime. The term “hallucinogen” describes LSD (lysergic acid diethylamide), LSA (d-lysergic acid amide), “magic” mushrooms, DOI (2,5-dimethoxy-4-iodoamphetamine), *Salvia divinorum*, ketamine, and/or mescaline. The term “opioid” describes heroin, methadone, opium, and recreational use of codeine, oxycodone, and/or buprenorphine. The term “inhalant” describes amyl nitrate and/or nitrous oxide. The term “sedative” describes GHB (or “Fantasy”) and/or recreational use of benzodiazepine or antidepressants.

**Table 3:** Summary of lifetime use of stimulants and cannabis in the stimulant group.

Subject	Total stimulants	Amphetamines	Ecstasy	Cannabis
1	2,241	2,072	169	28
2	833	832	1	13
3	828	402	426	3,675
4	367	211	156	4,380
5	332	228	104	1,251
6	213	3	210	120
7	209	208	1	6,570
8	199	93	106	23
9	156	3	153	1,529
10	57	5	52	4,380
11	38	26	12	5,616
12	36	10	26	474
13	31	3	28	876
14	27	26	1	270
15	22	2	20	1,456
16	19	8	11	6
17	19	1	18	15
18	18	—	18	153
19	17	4	13	2,763
20	16	10	6	1,092
21	12	3	9	72

Mean	271 ± 513	207 ± 483	73 ± 104	1,655 ± 2,061

Single-subject and mean data are presented (number of times used). The term “amphetamine” describes amphetamine and amphetamine-like drugs such as methamphetamine, cocaine, dexamphetamine, Ritalin, and pemoline (one subject). The term “ecstasy” describes ecstasy, MDA (3,4-methylenedioxyamphetamine, one subject), and MCAT (mephedrone, one subject).
